# Case report: *IL12RB1* deficiency in an infant with disseminated Bacille Calmette-Guérin disease

**DOI:** 10.3389/fimmu.2025.1728612

**Published:** 2026-01-26

**Authors:** Xiao Ma, Juping Chen, Junqiao Cheng, Jingbo Wang

**Affiliations:** 1 Preventive Care Department, Jinhua Maternity and Child Health Care Hospital, Jinhua, Zhejiang, China; 2 Jinhua Maternity and Child Health Care Hospital, Jinhua, Zhejiang, China

**Keywords:** Bacille Calmette-Guérin (BCG) vaccine, disseminated BCG disease, IL12RB1 mutation, immunology, mendelian susceptibility to mycobacterial disease

## Abstract

Disseminated Bacille Calmette-Guérin (BCG) disease is a rare adverse reaction to BCG vaccination. We report a neonate presenting with a progressive left axillary mass 3 months post-vaccination. High-throughput sequencing confirmed Mycobacterium bovis (BCG strain) infection. Genetic testing confirmed inborn errors of immunity (IEI) caused by interleukin (IL) 12 receptor β1 deficiency. An expert panel diagnosed BCG disease (acute hematogenous disseminated BCG disease and axillary lymph node involvement) due to the immunodeficiency. After about 28 months of anti-BCG combination therapy with interferon-gamma (IFN-γ), the infection was successfully controlled. The patient continues to receive maintenance therapy with only interferon-gamma (IFN-γ). The study explores the optimal timing and target populations for BCG vaccination in high TB burden countries to minimize severe adverse effects.

## 1 Introduction

Tuberculosis remains a major global public health threat. In 2023, the World Health Organisation estimated a worldwide tuberculosis incidence of 134 per 100,000 population, ranking it as the first leading cause of death from a single infectious agent (1). Since 1921, the Bacille Calmette–Guérin (BCG) vaccine has been the only available vaccine for tuberculosis prevention (2). Although generally safe, BCG vaccination can lead to adverse events, with an estimated incidence ranging from 100 to 1000 per million doses. Among these, disseminated BCG disease is one of the most severe complications, occurring in approximately 1.56 to 4.29 per million doses, and is associated with high mortality (3). This study presents a representative case of an infant who developed a progressively enlarging left axillary mass three months after BCG vaccination. Genetic testing confirmed inborn errors of immunity (IEI) with interleukin (IL) 12 receptor β1 mutation. We report the first Mendelian susceptibility to mycobacterial disease (MSMD) case caused by a novel *IL12RB1* compound heterozygous mutation, c.731C>A (p.S244X) at exon 8. This finding expands the known mutation spectrum of this gene. The diagnosis process of this case commenced with the clinical phenotype of BCG disease and proceeded to explore the underlying immunodeficiency background by genetic testing, thereby achieving precise disease tracing and personalized treatment.

## 2 Case presentation

**Figure 1:**
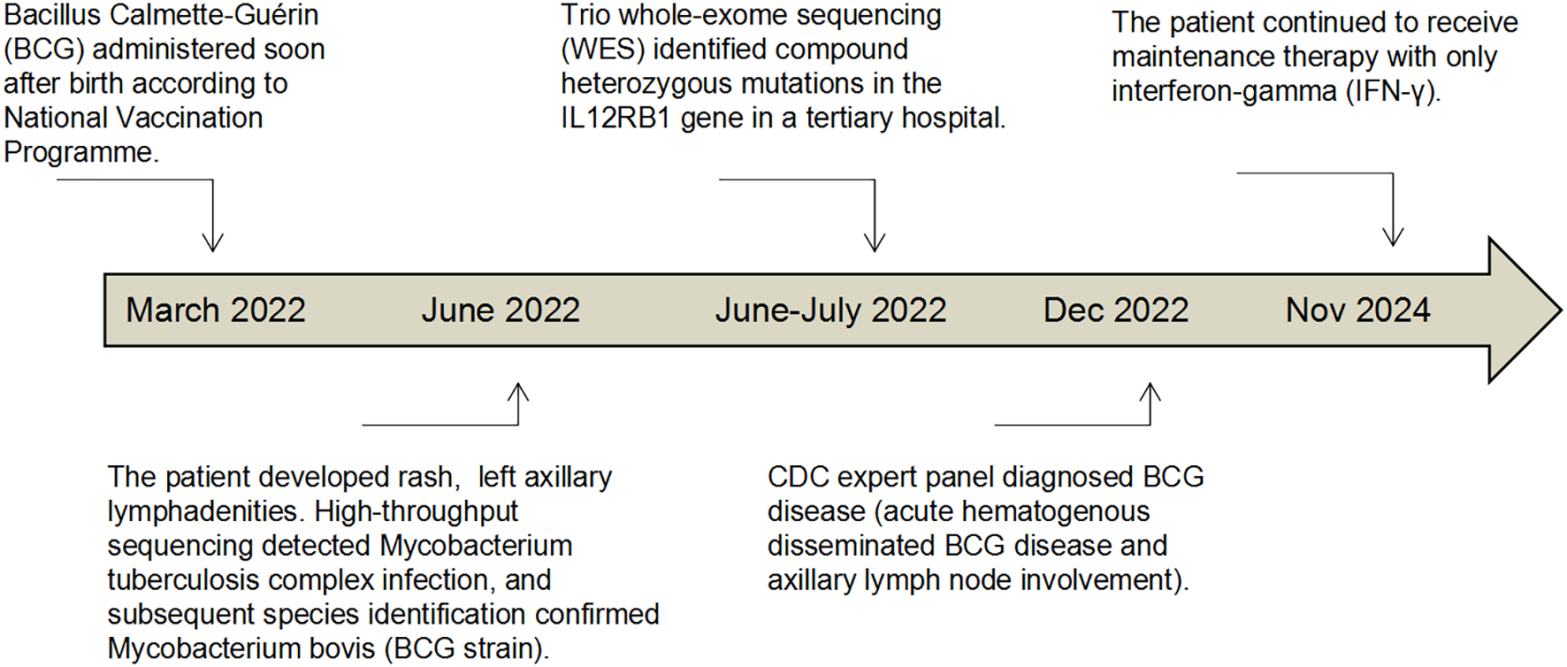
This figure shows the patient’s timeline of care and highlights the key dates and major interventions.

The patient was a full-term female infant delivered by spontaneous vaginal delivery, with a birth weight of 3200 g and an Apgar score of 10. Her parents denied any family history of hereditary diseases. According to the standard immunization schedule, she received 0.1 ml of the Bacillus Calmette-Guérin (BCG) vaccine and the first dose of the hepatitis B vaccine within 24 hours after birth. At three months of her age, she was initially seen at a local pediatric outpatient clinic for a “rash for 2 days”. Physical examination revealed polymorphic red maculopapular eruptions on the face and neck without exudation. She was re-evaluated the following day for a “red, swollen mass in the left axilla for 24 hours”. Examination showed an oval-shaped mass in the left axilla with marked tenderness (2+). The affected limb had a normal range of motion, and no systemic symptoms, such as fever, were present. Laboratory tests revealed an elevated C-reactive protein level of 29.78 mg/l and a white blood cell count of 17.67×10^9^/l (neutrophils 43.6%). Other inflammatory markers were within normal limits. Appropriate medical treatment directed at the infection and symptoms was provided. On day 4 of the illness, the patient returned with a low-grade fever (peak temperature 38.1°C). Axillary ultrasound revealed multiple enlarged lymph nodes, with the largest mass in the left axilla measuring approximately 6.0 cm × 4.0 cm. The local skin temperature was elevated, the infant exhibited marked crying upon palpation, and no fluctuation was detected. Due to the poor response to local treatment and progression of systemic inflammatory signs, the patient was hospitalized for further systemic evaluation and intensified therapy. The key clinical information was presented on a timeline (**Figure 1**).

## 3 Evaluation

Physical examination upon admission revealed a body temperature of 38.1°C. Laboratory results of blood count were presented in **Table 1**. On hospital day 3, an abscess incision and drainage procedure was performed. Acid-fast staining of the pus was positive (4+). Metagenomic Next-Generation Sequencing (mNGS) detected Mycobacterium tuberculosis complex infection, and subsequent species identification confirmed Mycobacterium bovis (BCG strain). Fourteen days after symptom onset, the patient was transferred to a tertiary hospital for further management. Examination at that time revealed a 0.5 × 0.5 cm inflammatory induration at the BCG inoculation site. The complete blood count, lymphocyte subset, immunoglobulin, and complement results from the patient’s second admission were shown in **Tables 1**–**3**, respectively. Immunological tests for tuberculosis (T-SPOT.TB and PPD skin test) were both negative. To investigate a potential underlying immunodeficiency, trio whole-exome sequencing (WES) was performed. The results identified compound heterozygous mutations in the *IL12RB1* gene: a maternally inherited mutation at Exon 8, c.731C>A (p.S244X), and a paternally inherited mutation at Exon 7, c.632 G>C (p.R211P).

**Table 1:** Hematological findings in the patient’s blood sample.

Description^1^	Value (first hospitalization)	Value (second hospitalization)	Normal range
TWBCs	21.89 × 10^9^/l	27.77 × 10^9^/l	4.3-14.2×10^9^/l
NEUT%	42.6%	25.6%	7.0-56.0%
Lym%	44.2%	58.6%	26.0-83.0%
RBCs	3.40×10^12^ /l	3.45×10^12^ /l	4.05-5.05×10^12^ /l
Hemoglobin	96 g/l	90 g/l	97-183 g/l
Platelets	609×10^9^/l	801×10^9^/l	183-614×10^9^/l
CRP	42.54 mg/l	14.84 mg/l	<6 mg/l
ESR	76 mm/h	99 mm/h	0-15 mm/h

^1^TWBCs, total white blood cells; Lym%, lymphocyte percentage; NEUT%, neutrophil percentage; RBCs, red blood cells; CRP, C-reactive protein; ESR, erythrocyte sedimentation rate.

**Table 2:** Patient lymphocyte subset flow cytometry results.

Description	%	Normal range	Absolute count	Normal range /μl
CD3	56.10	61.1-77.0	1173	470-3260
CD4	38.73	25.8-41.6	810	200-1820
CD8	16.94	16-20	354	130-1350
CD4/CD8	2.29	0.71-2.78	2.29	0.71-2.78
B cells (CD19)	33.58	7.30-18.20	702	50-670

**Table 3:** Patient immunoglobulin and complement results.

Description	Value	Normal range
Ig G	10.3 g/l	7.00-16.00 g/l
Ig A	0.31 g/l	0.70-4.00 g/l
Ig M	2.62 g/l	0.40-2.30 g/l
C3	1.60 g/l	0.90-1.08 g/l
C4	0.21 g/l	0.10-0.40 g/l
Ig E	101.00 IU/ml	<190 IU/ml

## 4 Treatment

Following a 7-day inpatient course of anti-BCG therapy (rifampicin 75 mg, isoniazid 75 mg, ethambutol 100 mg daily, and linezolid 55 mg three times daily), the patient was discharged upon clinical improvement. She was advised to continue anti-BCG combination therapy with interferon-gamma (IFN-γ) and undergo scheduled follow-up assessments.

## 5 Outcome and follow-up

A multidisciplinary CDC expert panel concluded that while the underlying *IL-12RB1* deficiency was not vaccine-related, the subsequent development of acute hematogenous disseminated BCG disease and axillary lymph node involvement was classified as an adverse reaction to BCG vaccination. The patient attended scheduled follow-ups (monthly until 6 months, then quarterly until 1 year), with additional visits as needed. At her follow-up in Nov. 2024, she was on interferon-gamma (IFN-γ) therapy only, with mild developmental delay but otherwise clinically stable.

## 6 Discussion

This study describes a novel *IL12RB1* mutation and highlights phenotypic variability. Although the paternal mutation in exon 7 (c.632G>C, p.R211P) has been previously reported in a case of recurrent Salmonella enteritidis D sepsis and pneumatocele (4), our patient presented with disseminated BCG disease, demonstrating a distinct clinical phenotype. In contrast, the maternal mutation in exon 8 (c.731C>A, p.S244X) is novel and has not been documented in the literature. As a serious immunization-related adverse event, disseminated BCG disease is often linked to underlying inborn errors of immunity (IEI). *IL12RB1* deficiency, a form of Mendelian susceptibility to mycobacterial disease (MSMD), disrupts the IL-12/IFN-γ signaling pathway (5, 6). This impairment affects IL-12 receptor function on NK and T cells, reducing IFN-γ production and compromising Th1 immunity. Consequently, the host loses the ability to clear BCG, leading to systemic dissemination (7).

According to the WHO, China remains a high tuberculosis burden country (1). In accordance with the recommendations of the BCG vaccination position paper, newborns should receive the BCG vaccine as soon as possible (8). To provide early immunization against severe tuberculosis like tuberculous meningitis, it is a national policy in China that all newborns without contraindications receive the BCG vaccine within 24 hours after birth. The BCG vaccine strain used in China is D2PB302, administered via intradermal injection at a dose of 0.1 ml (containing 0.05 mg of Mycobacterium bovis BCG) (9).

Nevertheless, the issue of the optimal timing for BCG vaccination deserves further discussion. IEIs often lack specific clinical manifestations in the neonatal period, making it difficult to identify individuals with underlying immune defects through routine health screening before vaccination. This represents a fundamental limitation in pre-vaccination risk assessment. Considerable variation exists in BCG vaccination policies across countries: in European nations with low tuberculosis incidence, only about 38% implement universal neonatal vaccination; a 2010 survey showed that 35.48% (11/31) of participating countries had completely discontinued routine BCG vaccination for all children (10). Importantly, the decline in BCG vaccination coverage in European and American countries has been accompanied by a concurrent decrease in tuberculosis incidence, suggesting that a targeted vaccination strategy focusing on high-risk populations may be feasible (11). Japan adjusted its vaccination strategy in 2005 by postponing the BCG vaccination age from within 6 months to 5–8 months (no later than 1 year of age), and further revised it in 2013 to vaccination under 1 year of age (12), aiming to allow a longer time window for IEI screening. Subsequent active surveillance data showed that the incidence of disseminated BCG disease was 1.3 per 100,000 doses among those vaccinated before 6 months of age, compared to 0.6 per 100,000 doses among those vaccinated at 6 months or older. Although no statistically significant difference was observed between the two groups—possibly due to the low incidence of disseminated disease and limited sample size—the data still suggest a potential safety advantage associated with delayed vaccination. Following the 2016 policy adjustment in the Taiwan province of China that delayed BCG vaccination from within 5 months to after 5 months of age, the incidence of BCG-induced osteomyelitis decreased from 41.4 to 7.9 cases per million, and no significant increase in tuberculous meningitis was observed, despite a subsequent decline in vaccination coverage to 53% (13, 14).

Appropriately delaying BCG vaccination may help extend the clinical observation window and facilitate early identification of immunodeficiency. However, in high tuberculosis burden areas, newborns still face significant risks from Mycobacterium tuberculosis infection. The WHO explicitly recommends that in regions with high tuberculosis prevalence, newborns should receive the BCG vaccine as soon as possible after birth, as this strategy demonstrates a favorable cost-effectiveness ratio. However, significant disparities in tuberculosis incidence exist among different provinces in mainland China. According to the data (15), the reported incidence of pulmonary tuberculosis in some provinces has fallen below 40 per 100,000 population. In such low-incidence settings, the public health benefits of widespread BCG vaccination may be outweighed by the potential risks. Therefore, a routine BCG vaccination strategy for all newborns immediately after birth may no longer be applicable in regions with lower tuberculosis incidence.

While BCG vaccination is safe for the general population and remains a cornerstone of neonatal immunization in many regions, policy adjustments may be warranted in low-incidence settings. However, in infants with specific IEI, BCG vaccination can lead to severe complications, and BCG disease may even serve as the initial clinical manifestation of underlying immune defects. Studies indicate that among Severe Combined Immunodeficiency (SCID) infants receiving BCG vaccination, approximately 51% develop related complications, with about two-thirds manifesting granulomatous disease and one-third presenting localized disease (16). Although individual IEI with BCG-osis conditions are rare, they are associated with significantly high mortality rates (17). In Singapore, following routine BCG vaccination shortly after birth and synchronous newborn screening for SCID, infants with abnormal results receive immediate anti-mycobacterial therapy (18). Accumulating evidence from multiple countries and regions confirms that routine immunological screening before BCG vaccination represents a necessary and effective public health strategy (19, 20).

In summary, while disseminated BCG disease is a rare complication of vaccination, it carries a grave prognosis, particularly in individuals with undiagnosed IEIs, with mortality rates ranging from 50% to 70% (21). These findings highlight the need to reevaluate clinical practice: in high tuberculosis burden countries, particular attention must be paid to the timing and targeting of BCG immunization. There is an urgent need to develop and implement clinically feasible strategies for early identification of infants at risk of immunodeficiency—such as through family history assessment, newborn screening, or rapid immunologic testing—before vaccine administration. Enhancing clinical vigilance and adopting a more individualized approach to BCG vaccination may significantly reduce the incidence of these severe adverse events and improve overall safety outcomes in national immunization programs.

## Data Availability

The original contributions presented in the study are included in the article/supplementary material. Further inquiries can be directed to the corresponding author.
